# Natural cycle increases the live-birth rate compared with hormone replacement treatment for frozen-thawed single euploid blastocyst transfer

**DOI:** 10.3389/fendo.2022.969379

**Published:** 2022-10-28

**Authors:** Xiaofang Li, Yan’e Gao, Juanzi Shi, Wenhao Shi, Haiyan Bai

**Affiliations:** ^1^ Assisted Reproduction Center, Northwest Women’s and Children’s Hospital, Xi’an, Shaanxi, China; ^2^ Department of Gynecology and Obstetrics, The Second Affiliated Hospital, Xi’an Jiaotong University, Xi’an, Shaanxi, China

**Keywords:** frozen embryo transfer, hormone replacement treatment, natural cycles, live birth rate, preimplantation genetic testing

## Abstract

**Background:**

A number of studies have compared the clinical outcomes between the two endometrial preparation methods: natural cycles (NCs) and hormone replacement treatment (HRT) before frozen embryo transfer, but the results were conflicting. In order to mitigate the potential effect of embryos per se, several researchers have worked on this subject for euploid blastocyst transfer, but the results were still inconsistent. Therefore, the present study was aimed to investigate the clinical outcomes between HRT and NC for autologous single vitrified–warmed euploid blastocyst transfer based on our data.

**Methods:**

A total of 598 frozen-thawed single euploid blastocyst transfer cycles in the assisted reproductive center of Northwest Women’s and Children’s Hospital from January 2014 to May 2021 were retrospectively analyzed. Women were stratified into the NC (n = 125) or HRT (n = 473) group according to the patient’s preference and the physician’s guidance. Multivariate regression models and subgroup analysis were constructed to analyze the association between endometrial preparation and live birth.

**Results:**

Women in the NC group had a higher live birth rate (68.80% versus 58.35%, *P* = 0.034) and a lower risk of total pregnancy loss (8.51% versus 21.14%, *P* = 0.005) when compared with women in the HRT group. The biochemical pregnancy rate (75.20% versus 74.00%, *P* = 0.784) and clinical pregnancy rate (74.40% versus 69.98%, *P* = 0.334) were similar between the two groups (NC versus HRT). NC was associated with an increased odds of live birth compared with HRT by different multivariable analysis models (Model 1: adjusted odds ratio [aOR], 95% confidence interval [CI]: 0.57, 0.36 - 0.90; Model 2: aOR, 95%CI: 0.57, 0.35 - 0.92). In addition, the increased chance of live birth in the NC group was found in all subgroups. No major obstetrical complications and two malformation livebirths were reported.

**Conclusions:**

In women undergoing single euploid frozen blastocyst transfers, the NC group was associated with a lower pregnancy loss rate and an ultimately higher live birth rate than the HRT group. Although HRT is convenient for both clinicians and patients, the lower live birth rate should be taken into account and NC might be the first choice of endometrial preparation method.

## Introduction

With the improvements in embryo cryopreservation techniques, the proportion of frozen embryo transfer (FET) has increased dramatically in the past decades. FET becomes an indispensable part of assisted reproduction technology (ART) area owing to its advantages such as fertility preserving, preimplantation genetic testing (PGT), premature progesterone rising, and avoiding the ovarian hyperstimulation syndrome. In addition, single blastocyst transfer enables to reduce the risk of multiple pregnancy and associated complications without compromising the clinical outcome. Therefore, most centers use vitrified single-blastocyst transfer in most FET cycles.

In order to synchronize the endometrium and embryo before FET, various methods for endometrial preparation have been explored. The classic regimens are hormone replacement treatment (HRT) and natural cycles (NCs) (1). In the HRT, estrogen should be supplemented for the growth of follicles and promoting the proliferation of endometrium. However, the HRT cycles may increase cost and potential complications such as thrombotic disorders. In the NC, although no estrogen is administered, women should be monitored frequently and hold a higher cancellation rate (2). Since each protocol has its pros and cons, clinical outcomes should be one of the important evaluation criteria to determine the optimal method.

A number of retrospective studies and prospective randomized controlled trials (RCTs) have compared the clinical outcomes between the two endometrial preparation methods (NC and HRT) before FET, but the results were conflicting (3–5). Groenewoud et al. demonstrated that no significant difference was found in the live birth rate between HRT and NC cycles by an RCT (3). In 2017, Ghobara et al. published a Cochrane review, also concluding that no superiority of one method was found over the other, which was consistent with the conclusion of a systematic review and meta-analysis by Groenewoud et.al (6, 7). Morozov et al. and Fernanda et al. reported that NC cycles were associated with better clinical outcomes in comparison to HRT cycles (8, 9). Conversely, Zheng et al. and Hill et al. found that NC cycles had a decreased live birth rate in comparison to HRT cycles (10, 11). In order to mitigate the potential effect of embryos per se, several researchers have worked on this subject for euploid blastocyst transfer, but the results were still inconsistent (12, 13). In view of these controversial results, we investigated the live birth rate and perinatal outcomes between an NC and HRT in preparation for euploid, vitrified–warmed blastocyst transfer based on our data.

## Materials and methods

### Ethical approval and study population

Records were extracted and retrospectively reviewed for women who underwent a euploid, vitrified–warmed blastocyst transfer between January 2014 and May 2021 at the reproductive center of Northwest Women’s and Children’s Hospital in the People’s Republic of China. The current study was approved by the ethics committee of the hospital (number 2021002), and written informed consent was obtained from each patient before PGT treatment. Due to the retrospective nature, the informed consent of the present study was waived.

Women were included if they met the following criteria (1): women who had previous PGT cycles with euploid blastocyst cryopreservation; (2) women with endometrial thickness ≥7 mm before the start of progesterone administered; (3) women with regular menstrual cycles (21–35 days); (4) women who underwent HRT-FET or NC-FET of a single autologous blastocyst; and (5) the type of insemination was intracytoplasmic sperm injection (ICSI). Exclusion criteria included (1) women using blastocyst derived from vitrified and/or donor oocytes for fertilization; (2) women with other factors affecting pregnancy such as endometriosis, intrauterine adhesion, uterine cavity effusion, and untreated hydrosalpinx; (3) FET cycles with GnRH-agonist pretreatment; (4) PGT performed on previous vitrified embryos; (5) mosaic blastocyst transfer cycles; and (6) cycles with missing data and women lost to follow-up.

The baseline characteristics of the study population were collected, including a woman’s age at oocyte retrieval or embryo transfer (years), a man’s age at oocyte retrieval (years), BMI (weight (kg)/[height (m)]2), baseline FSH (bFSH, FSH value on day 2–day 4 of menstrual period), the number of antral follicle counts (AFC), gravidity (0, 1, or ≥2), parity (0, 1, or ≥2), and years of infertility. Cycle characteristics included indications for PGT (recurrent spontaneous abortion, history of abnormality of chromosome of fetus, chromosomal structural abnormality, monogenic disorder, or others), the protocol of controlled ovulation hyperstimulation (COH) in the fresh cycle (agonist or antagonist), the number of oocytes retrieved in the fresh cycle, the transfer rank of FET cycles, endometrial thickness before progesterone administration (Em), the triple-line endometrial pattern, and the day and quality of embryo development at transfer.

### in vitro fertilization (IVF) and preimplantation genetic testing procedures

The COS protocols, laboratory procedures, and luteal phase support were fully described in previous publications. When at least three follicles’ diameter ≥18 mm, human chorionic gonadotrophin (hCG) would be administered to induce follicular maturation. Oocytes were retrieved under transvaginal ultrasound (TVS) guidance 36 h later. Hyaluronidase was used to remove the granulosa cells after incubating for 3–4 h; ICSI was performed then. Embryos were cultured to D5 or D6. The blastocysts formed after the afternoon of the D6 were all discarded. Non-contact laser was used to perform biopsy on fully expanded blastocysts. A total of 8–10 trophoblast ectoderm cells were removed. Next-generation sequencing was used to assess the biopsies according to the manufacturer’s instructions [ChromInstTM; Xukang Medical Science & Technology (Suzhou) Co., Ltd]. Quantifying the library pool by quantitative real-time PCR before sequencing and then sequencing was performed on the Illumina next-generation sequencing platform. After biopsy, all blastocysts were vitrified immediately awaiting PGT results.

### Preparation of endometrium

Women were assigned to different endometrial preparation groups according to the patients’ preference or schedule or the habitual practice of different physicians.

In the NC group, women underwent a urine hCG exam on days 8–12 of their menstrual cycle first. Then, we monitored follicle sizes using TVS. When the mean diameter of patients’ leading follicle on TVS reached 15 mm, they need to come to the hospital daily for TVS testing. Sometimes, serum hormone levels like luteinizing hormone (LH), progesterone, and estrogen were tested when the dominant follicle had reached a mean diameter of >17 mm to assess the time of ovulation. Ovulation was confirmed by TVS, and then, the intramuscular injection of progesterone was administered (20 mg per day for 1 day and then 40 mg per day for 4 days). Blastocyst transfer was scheduled on D5 if the day of ovulation was D0. From the day of blastocyst transfer, a combination of oral progesterone (20 mg per day, dydrogesterone, Duphaston; Abbott Biologicals B.V., Olster, Netherland) and the intramuscular injection (IM) of progesterone (40 mg daily, Zhejiang Xianju Pharmaceutical Co., Ltd., Zhejiang, China) was administered for luteal phase support, which was continued for 10 weeks of gestation. The dose of progesterone was tapered every 3 days from the 10th gestational week (such as IM: 40 mg ≥ 20 mg; oral: 20 mg ≥ 10 mg).

In the HRT group, women also took a urine human chorionic gonadotropin (hCG) test and a TVS on the fifth day of menstruation first. Then, oral estradiol valerate tablets (4–6 mg/d, Bayer, Leverkusen Germany) were given according to the endometrium thickness of the previous fresh cycle. Vaginal ultrasound was performed 5–10 days later to measure the endometrium thickness. If the endometrium thickness reached 7 mm or more, the intramuscular injection of progesterone was administered (20 mg per day for 1 day, 40 mg per day for 2 days, and then, 60 mg per day for 3 days). The transfer was cancelled if the serum progesterone value was more than 1.5 ng/ml. Blastocyst transfer was scheduled on D6 if the day commencing progesterone was D0. In addition to continuing the intramuscular injection of progesterone (60 mg daily) and estrogen, 20 mg of dydrogesterone was added daily until 10 weeks of gestation. Blood hCG was measured 12 days after transfer. The dose of estrogen was tapered by approximately one-third every 3 days in case biochemical pregnancy was confirmed by the hCG test. The dose of progesterone was tapered every 3 days from the 10th gestational week (such as IM: 60 mg ≥ 40 mg ≥ 20 mg; oral: 20 mg ≥ 10 mg).

### Clinical outcome measure

Biochemical pregnancy was defined as a serum β-hCG level >20 IU/L at the 12th day after blastocyst transfer. Clinical pregnancy defined that gestational sac was determined by ultrasonographic at the sixth-to-eighth week of gestation. Early pregnancy loss was defined as spontaneous abortion before 13 weeks of pregnancy or no gestational sac was confirmed after biochemical pregnancy. Live birth was defined as the delivery of a live-born baby beyond 22 weeks of gestation. Delivery and neonatal outcomes were also analyzed in the present study, including the sex ratio of live births, gestational age, preterm delivery (a birth that takes place between 22 and 37 weeks of gestational age), mean birth weight (g), and delivery mode (caesarian section or natural delivery). For perinatal outcomes, we restricted the analysis to singletons to rule out the influences of multiple pregnancies.

### Data analysis and statistics

All data were exported from the electronic medical record system; analyses were performed using IBM^®^SPSS^®^ software (version: 22.0, SPSS Inc. Headquarters, USA) and the statistical packages R (The R Foundation; http://www.r-project.org; version 3.4.3) and EmpowerStats (www.empowerstats.com; version: 3.0, X&Y Solutions Inc.). Continuous variables were displayed as the mean ± SEM, and categorical variables were presented as frequencies (percentages). The Pearson χ^2^ test or Fisher’s exact test was used for qualitative data, and the Kruskal–Wallis test was used for quantitative data. Logistic regression analysis was used to identify the possible confounders and independent factors that may modify the odds of live birth. Interaction and stratified analyses were performed according to women’s age at embryo transfer (<28, 29–31, and ≥32 years old), the number of AFCs (<11, 11–15, and ≥16), the protocol in the fresh cycle (agonist and antagonist), Em (<9.4, 9.5–9.9, and ≥10.0 mm), the triple-line endometrial pattern (A, B, and C), the transfer rank (1, 2 and ≥3), the number of oocytes retrieved (<10, 10–14, and ≥15), the day of embryo development at transfer (day 5 and day 6), and embryo quality (lower quality and good quality). The differences were considered as statistically significant at a *P* value < 0.05.

## Results

Our study recruited 598 single frozen-blastocyst transfer cycles. During the study period, 479 cycles were the first FET cycle. The remaining 119 cycles were women who received multiple frozen-thawed euploid blastocyst transfers, or received non-PGT FET at least once before euploid blastocyst transfer. For all the frozen euploid blastocyst transfers, the biochemical pregnancy rate, clinical pregnancy rate, live birth rate, and early clinical loss (per clinical pregnancy) were 74.25%, 70.90%, 60.54%, and 11.32%, respectively.

### Characteristics of the patients and assisted reproduction technology cycles

As shown in **Table 1**, there were no statistical differences among the NC and HRT groups in the mean women’s age at retrieval and transfer, the mean men’s age, the BMI, infertile years, baseline FSH, gravidity, PGT indication, the number of transfer cycles, the triple-line endometrial pattern, and the day and quality of blastocyst development at transfer. The AFC in the HRT group was slightly higher when compared with the NC group (13.97 ± 5.60 versus 12.04 ± 4.74, *P* < 0.001). The endometrial thickness was thinner in the HRT group when compared with the NC group (9.83 ± 1.34 versus 10.74 ± 1.57, *P* < 0.001). The NC group had a lower proportion of nullipara patients when compared with the HRT group (80.00% versus 89.01%, *P* = 0.024). Women in the HRT group had higher oocytes retrieved in the fresh cycle when compared with women in the NC group (12.54 ± 6.33 versus 13.80 ± 7.16, *P* = 0.042). The protocols used in the fresh cycle were s significantly different in the two groups; the proportion of the agonist protocol in the fresh cycle was higher in the HRT group when compared with the NC group (48.84% versus 33.60%). Accordingly, patients in the NC group had a significantly higher proportion of the antagonist protocol in the fresh cycle when compared with women in the HRT group (66.40% versus 51.16%) (*P* = 0.002).

**Table 1:** Characteristics of the patients and assisted reproduction technology cycles (mean ± SEM or n%).

Variables	NC	HRT	*P*-value
n	125	473	
Women’s age at oocyte retrieval	30.54 ± 3.54	30.27 ± 3.69	0.361 ^KW^
Women’s age at embryo transfer	30.89 ± 3.45	30.62 ± 3.71	0.354 ^KW^
Male’s age at embryo transfer	32.38 ± 3.93	31.94 ± 4.16	0.262 ^KW^
BMI	22.26 ± 2.98	22.31 ± 3.35	0.768 ^KW^
Infertile years	2.31 ± 2.82	2.28 ± 2.15	0.149 ^KW^
Ovarian reserve function			
bFSH	6.94 ± 2.15	6.84 ± 2.29	0.231 ^K^
AFC	12.04 ± 4.74	13.97 ± 5.60	**< 0.001 ^K^**
Gravidity			0.253 ^K^
0	40 (32.00)	180 (38.05)	
1	27 (21.60)	76 (16.07)	
≥2	58 (46.40)	217 (45.88)	
Parity			**0.024 ^K^**
0	100 (80.00)	421 (89.01)	
1	24 (19.20)	48 (10.15)	
≥2	1 (0.80)	4 (0.85)	
PGT indication			0.215 ^K^
RSA	16 (12.80)	77 (16.28)	
History of abnormality of chromosome of fetus	4 (3.20)	38 (8.03)	
Chromosomal structural abnormality	85 (68.00)	295 (62.37)	
Monogenic disorder	10 (8.00)	25 (5.29)	
Others	10 (8.00)	38 (8.03)	
Protocol in fresh cycle			**0.002 ^K^**
Agonist	42 (33.60)	231 (48.84)	
Antagonist	83 (66.40)	242 (51.16)	
Number of oocytes retrieved	12.54 ± 6.33	13.80 ± 7.16	**0.042 ^KW^**
Transfer rank			0.443 ^K^
1	105 (84.00)	374 (79.07)	
2	15 (12.00)	70 (14.80)	
≥3	5 (4.00)	29 (6.13)	
Em (mm)	10.74 ± 1.57	9.83 ± 1.34	**< 0.001 ^KW^**
Triple-line endometrial pattern			0.529 ^K^
A	15 (12.00)	45 (9.51)	
B	105 (84.00)	400 (84.57)	
C	5 (4.00)	28 (5.92)	
Day of embryo development at transfer			0.693 ^K^
5	105 (84.00)	404 (85.41)	
6	20 (16.00)	69 (14.59)	
Embryo quality			0.959 ^K^
Lower-quality embryo	75 (60.00)	285 (60.25)	
Good-quality embryo	50 (40.00)	188 (39.75)	

BMI, body mass index; AFC, antral follicle count; FSH, follicle-stimulating hormone; Em, endometrial thickness before progesterone administration; PGT, preimplantation genetic testing; RSA, recurrent spontaneous abortion; RIF, repeated implanting failure; A good-quality embryo was defined as a B4, B5, or B6 embryo ≥ BB (AA, AB, BA, BB) according to the grading scale proposed by Gardner; KW, Kruskal–Wallis test; K, Pearson’s chi-square test. Significant difference values are in bold.

### Assisted reproduction technology outcomes

No differences were found between the two groups in terms of the biochemical pregnancy rate (75.20% versus 74.00%, *P* = 0.784), clinical pregnancy rate (74.40% versus 69.98%, *P* = 0.334), late clinical loss rate (2.15% versus 2.42%, *P* = 1.000), and ectopic pregnancy rate (2.15% versus 0.60%, *P* = 0.196) (NC group versus HRT group, respectively). Women in the HRT group had a higher chance of early pregnancy loss when compared with women in the NC group (13.60 versus 3.23%, *P* < 0.05). The total pregnancy loss rate was also significantly higher in the HRT group versus the NC group. (21.14 versus 8.51%, *P* < 0.05). A significantly lower chance of live birth was found in the HRT group versus the NC group (58.35 versus 68.80%, *P* < 0.05) (**Table 2**).

**Table 2:** Comparison of clinical outcomes after frozen embryo transfer between patients who have undergone a natural cycle (NC) or hormone replacement treatment (HRT).

	NC	HRT	OR (95% CI) (NC as reference)	*P-* value
Cycles	125	473		
Primary outcome				
Live birth (per transfer)	86 (68.80)	276 (58.35)	0.64 (0.42, 0.97)	**0.034**
Secondary outcomes				
Biochemical pregnancy (per transfer)	94 (75.20)	350 (74.00)	0.94 (0.60, 1.48)	0.784
Clinical pregnancy	93 (74.40)	331 (69.98)	0.80 (0.51, 1.25)	0.334
Early clinical loss (per clinical pregnancy)	3 (3.23)	45 (13.60)	4.72 (1.43, 15.55)	**0.005**
Late clinical loss (per clinical pregnancy)	2 (2.15)	8 (2.42)	1.06 (0.22, 5.10)	1.000
Ectopic pregnancy (per clinical pregnancy)	2 (2.15)	2 (0.60)	0.26 (0.04, 1.88)	0.196
Total pregnancy loss (per biochemical pregnancy)	8 (8.51)	74 (21.14)	2.88 (1.34, 6.22)	**0.005**

Significant difference values are in bold. OR, odds ratio; CI, confidence interval.

### Association of endometrial preparation protocols with live birth

The endometrial preparation method was associated with live birth in the univariate analysis (OR = 0.64, 95% CI 0.42–0.97, *P* = 0.034) and multivariate analysis models 1 (OR = 0.57, 95% CI: 0.36 − 0.90, *P* = 0.016) and 2 (OR = 0.57, 95% CI: 0.35 − 0.92, *P* = 0.023) (**Table 3**). The results of interaction and stratified analyses are presented in **Supplemental Figure 1**. In terms of the live birth rate, significant between-group (NC and HRT) differences were found with women with the number of AFCs ≥16, women who used the agonist protocol in the fresh cycle, women with endometrial thickness before progesterone administration ≥10.0 mm, women who have undertaken the first FET cycle, women with the number of oocytes retrieved between 10 and 14, and women with day 5 embryo transferred and good-quality embryo transferred. A higher chance of live birth was observed in the NC group in all subgroups, and no significant interactions were found in any of the subgroups (*P* > 0.05 for all comparisons).

**Table 3:** Correlation between live birth and endometrial preparation (NC or HRT) in different models.

	Crude Model		Adjusted Model 1		Adjusted Model 2	
	OR (95% CI)	*P*-value	OR (95% CI)	*P*-value	OR (95% CI)	*P*-value
NC	Reference		Reference		Reference	
HRT	0.64 (0.42, 0.97)	**0.034**	0.57 (0.36, 0.90)	**0.016**	0.57 (0.35, 0.92)	**0.023**

Crude model: we did not adjust for any covariates.

Adjusted Model I: we adjusted for women age at retrieval, BMI, and embryo quality.

Adjusted Model II: we adjusted for women age at retrieval and embryo transfer, gravidity, parity, Em, triple-line endometrial pattern, embryo quality, day of embryo development at transfer, BMI, bFSH, PGT indication, transfer rank, protocol in the fresh cycle, and number of oocytes retrieved.

OR, odds ratio; CI, confidence interval.

Significant difference values are in bold.

### Neonatal and delivery outcomes

Of the 362 women who achieved newborns, 358 women had singletons and 4 women had twins. Neonatal and delivery outcomes in terms of the sex ratio, mean birth weight, mean length, and gestational age at delivery were comparable among the two groups. The proportion of the caesarian section was significantly higher in the HRT group than the NC group (75.91% versus 64.29%, *P* = 0.035). There was no neonatal death in the two groups (**Supplemental Table 1**). One strephenopodia malformation infant in the HRT group and one soft palate cleft infant in the NC group were reported. No major obstetrical complications were reported.

## Discussion

A normal embryo and a receptive endometrium are two essential elements for a live birth. PGT is used to select euploid blastocysts for transfer, which could improve the clinical outcomes of recurrent pregnancy loss related to the chromosomal factors of the embryo (14). However, the transfer of euploid blastocysts did not improve pregnancy outcomes in all women (15, 16). In this context, researchers have focused on the receptivity of the endometrium. The optimal scheme of endometrial preparation has been discussed a lot, but the conclusions were conflicting and most of the hitherto studies have focused on unbiopsied embryos (3, 11, 17). Our present study suggested that for the frozen transfer of euploid blastocysts, the live birth rate of HRT cycles was significantly lower when compared to NC cycles by univariate and multivariate analyses. The lower chance of live birth in the HRT group was also found in all subgroups considered and after careful adjustments.

As discussed before, although many literatures have suggested comparable outcomes between HRT and NC cycles, the results about the optimal endometrial preparation protocol have been conflicting (6, 7, 18). It is not easy to decipher the contradictions as many factors such as the heterogeneity in protocols, study populations, and technology levels in different periods may affect the outcomes. In addition, most of them were non-PGT cycles. In the present study, we only included euploid blastocyst transfers to eliminate the embryonic factor and assess the clinical outcome between HRT and NC cycles. A retrospective study of 12,950 FET cycles published by Li et al. concluded a comparable clinical pregnancy rate, higher pregnancy loss rate, and lower live birth rate when HRT cycles were compared with NC cycles, which were in line with our results and also consistent with a latest multicenter cohort study published by Vinsonneau et al. (19, 20).

Melnick et al. and Wang et al. have reported a better live birth rate in the NC group when compared with the HRT group for euploid blastocyst transfers, with sample sizes ranging from 113 to 389, which was consistent with our result (13, 21). In the study by Melnick et al. and Wang et al., the implantation and clinical pregnancy rates were lower, and, ultimately, the live birth rate was lower in the HRT group when compared with the NC group. However, the miscarriage rates were similar in the two groups. Due to the alterable width of the window of implantation, different centers have their own preferences for scheduling the transfer day. In our center, we schedule blastocyst transfer on D6 for HRT cycles if the day commencing progesterone was D0, which was different with Melnick et al.’s study. In addition, we have different study populations. These may cause the difference between the two studies and the current study. Conversely, Jin et al. concluded an equivalent live birth rate between NC and HRT in frozen euploid blastocyst transfers (12). In our study, we excluded GnRHa downregulation treatment cycles that might increase the clinical outcome and create a bias when compared with the NC group (16, 22). We also have different experiences on scheduling the transfer day. In both NC and HRT cycles, we schedule transfer 1 day later than Jin et al.’s and, ultimately, we have better live birth rates in both NC and HRT cycles than theirs (NC: 68.8% versus 50%; HRT: 58.35% versus 47.61%). On the other hand, we have different populations (catalogue of PGT indications, average age of female and women from different areas, and so forth). Finally, we concluded that the HRT group has a higher pregnancy loss rate and a lower live birth rate than the NC group with a similar sample size.

In many studies with frozen unbiopsied embryo transfers, the HRT cycles have a higher miscarriage rate than NC cycles, which were in accordance with our results (17, 23, 24). Nakamura et al. have worked on the morphology of the placenta, demonstrating that long-lasting structure changes in the placenta in HRT cycles and further cause the underdevelopment of the decidual layer (25). Patel et al. concluded that supraphysiologic estrogen levels have a negative impact on placentation through the increased apoptosis of trophoblast cells, which may contribute to pregnancy loss (26). On the other hand, since the absence of the corpus luteum in the HRT cycles, the first trimester pregnancy is entirely reliant on exogenous progesterone supplementation. Inadequate progesterone administration may hamper pregnancy outcomes. HRT increases the incidence of thromboembolic events and the risk of preeclampsia, which also leads to higher odds of pregnancy loss (27, 28).

The major strength of our study is that we included women who have undergone single euploid blastocyst transfer to eliminate the potential effect of embryos per se and focus on the receptivity of the endometrium in HRT and NC cycles. Another strength is that we took into account the effects of several factors like age, BMI, embryo quality, ovarian reserve function, transfer rank, and the indication of PGT; meanwhile, multivariate and subgroup analyses were also conducted to make the conclusion more reliable. On the other hand, we only included freeze-only transfer cycles to avoid the bias of the transferring of the highest-quality embryos in the fresh cycle. In addition, our data was real world-based, thus avoiding the bias of clinical trials. Furthermore, with the increasing of FET cycles, our present study has widespread clinical applicability.

This study does have some weaknesses. Due to the nature of the retrospective study, we could not adjust other potential confounders, such as the smoking status and the adjustment of clinicians’ preference and opinions during the study period. On the other hand, the women were allocated to the HRT or NC group according to clinical practice, which might create a selection bias. However, we only included women with regular menstrual cycles to try to minimize the selection bias, and we conducted multivariate analyses to control the confounding factors. Subgroup analysis also indicated that the results were stable. Since the average age of the current study population is approximately 30 years (range: 22–44), the readers should be cautious to extrapolating to patients older than 44 years of age. In our present study, a higher caesarian section rate in the HRT group was also observed, which agrees with the results of several pervious studies (19, 29). The higher risk of the low birth weight of live births was not found based on our data, which was consistent with Cerrillo et al.’s study but was contrary to Li et al.’s (17, 19). Therefore, more data were needed to confirm the relationship between perinatal outcomes and endometrial methods. Lastly, the study only included a single-center population with a relatively small sample size in China. In the future, more centers and larger sample sizes are still needed to verify the results of this study.

## Conclusions

The current study concluded that NC cycles have lower odds of pregnancy loss and ultimately higher odds of live birth than HRT cycles through multivariate regression analysis. Although HRT is convenient for both clinicians and patients, the lower live birth rate should be taken into account and NC might be the first choice of endometrial preparation method. Further RCTs are warranted to confirm the findings of the current study.

## Data availability statement

The raw data supporting the conclusions of this article will be available from the corresponding author, without undue reservation.

## Ethics statement

Written and informed consent was obtained from each patient for intracytoplasmic sperm injection/PGT-A. Informed consent for the present analysis was waived due to the nature of the retrospective study.

## Author contributions

HB, WS, and XL made a substantial contribution to the concept and design of the study; XL did the analysis and interpretation of data and drafted the article; JS and YG revised the manuscript critically for important intellectual content. All authors read and approved the final manuscript.

## Funding

This study was supported by the Project of Clinical Research Center for Gynecological Diseases of Shaanxi Province.

## Acknowledgments

The authors wish to thank all the staff from Northwest Women’s and Children’s Hospital for their assistance with the data collection, and we thank all the patients in this study.

## Conflict of interest

The authors declare that the research was conducted in the absence of any commercial or financial relationships that could be construed as a potential conflict of interest.

## Publisher’s note

All claims expressed in this article are solely those of the authors and do not necessarily represent those of their affiliated organizations, or those of the publisher, the editors and the reviewers. Any product that may be evaluated in this article, or claim that may be made by its manufacturer, is not guaranteed or endorsed by the publisher.

## Supplementary material

Supplementary Figure 1 Effect of endometrial preparation methods on live birth rate in each subgroup. Note: CI = confidence interval; OR = odds ratio; ^a^ Adjusted for female age at oocyte retrieval, BMI, infertile years and bFSH except the subgroup variable.
