# Supplementary figures and images for: Natural cycle increases the live-birth rate compared with hormone replacement treatment for frozen-thawed single euploid blastocyst transfer
Source: Front Endocrinol (Lausanne). 2022 Oct 28;13:969379. doi: 10.3389/fendo.2022.969379 (PMC9650322; doi:10.3389/fendo.2022.969379)

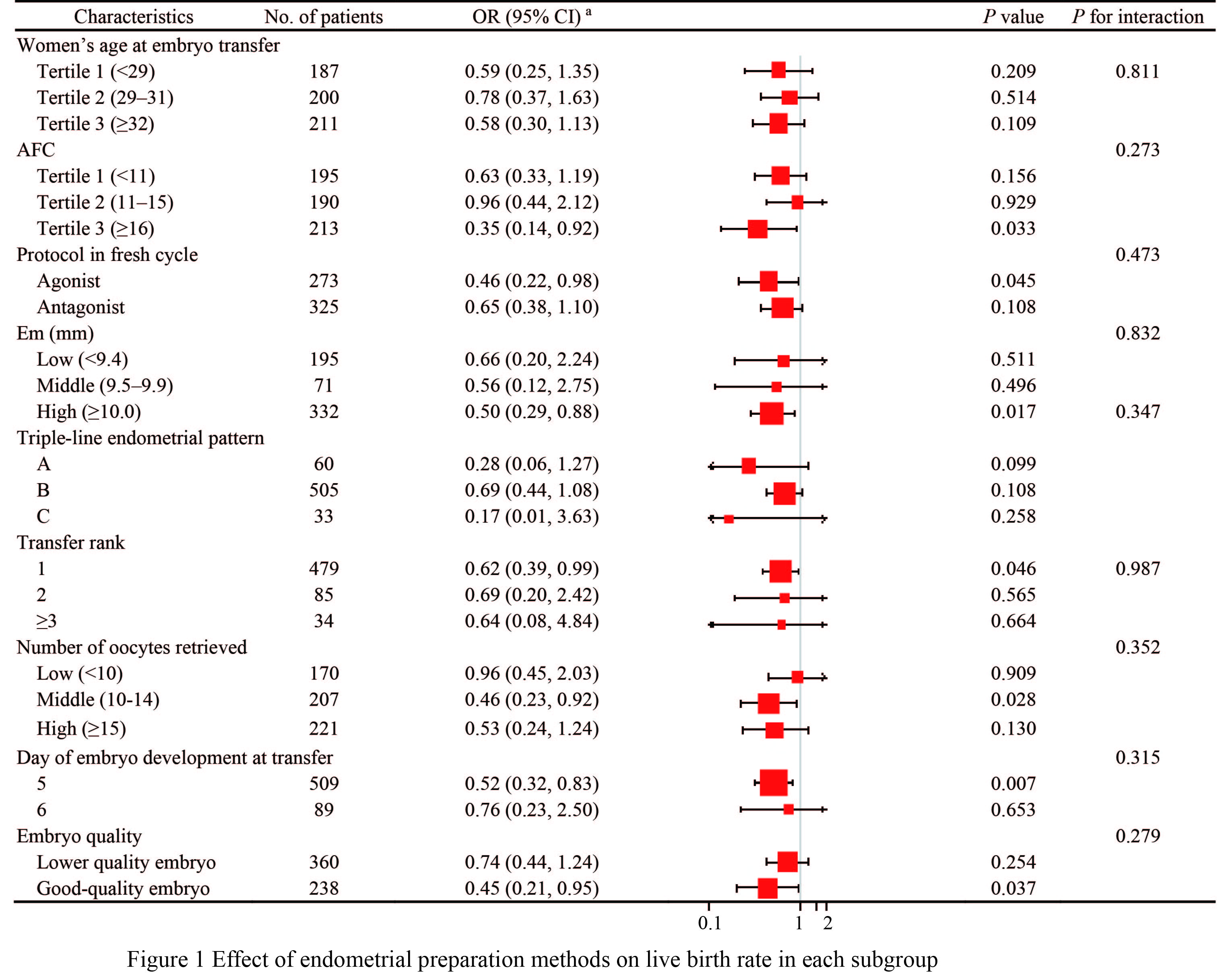

Supplement: Supplementary Figure 1 — Effect of endometrial preparation methods on live birth rate in each subgroup. Note: CI = confidence interval; OR = odds ratio; a Adjusted for female age at oocyte retrieval, BMI, infertile years and bFSH except the subgroup variable. [file Image_1.jpeg]
