# Supplementary material for: Natural cycle increases the live-birth rate compared with hormone replacement treatment for frozen-thawed single euploid blastocyst transfer
Source: Front Endocrinol (Lausanne). 2022 Oct 28;13:969379. doi: 10.3389/fendo.2022.969379 (PMC9650322; doi:10.3389/fendo.2022.969379)
Supplement: Supplementary file 2 [file Table_1.docx]

**Supplemental Table 1 Obstetrical, delivery and neonatal outcomes.**

|  | NC | HRT | *P* value |
| --- | --- | --- | --- |
| Cycles | 125 | 473 |  |
| Live birth (per transfer) | 86 (68.80) | 276 (58.35) | **0.034** |
| Singleton | 84 | 274 |  |
| Twin | 2 | 2 |  |
| Sex ratio (female: male) | 1: 1.211 | 1: 0.930 | 0.291 |
| Gestational age (days) | 273.04 ± 10.95 | 272.66 ± 12.57 | 0.991 |
| Preterm deliveries (22-37 wks) (% per live birth) | 4 (4.76) | 22 (8.03) | 0.313 |
| Mean birth weight (g) | 3423.87 ± 556.37 | 3367.48 ± 519.50 | 0.561 |
| <2500 g | 4 (4.76%) | 14 (5.11%) | 0.899 |
| ≥2500 g | 80 (95.24%) | 260 (94.89%) |  |
| Mean length(cm) | 50.13 ± 1.85 | 50.05 ± 1.82 | 0.562 |
| Caesarian section | 54 (64.29%) | 208 (75.91%) | **0.035** |

Note: Significant difference values are in bold. Significant difference values are in bold.
